# Supplementary figures and images for: Assessment of front and back of pack nutrition labels of selected convenience food products and snacks available in the Indian market
Source: PLoS One. 2024 Dec 6;19(12):e0314819. doi: 10.1371/journal.pone.0314819 (PMC11623555; doi:10.1371/journal.pone.0314819)

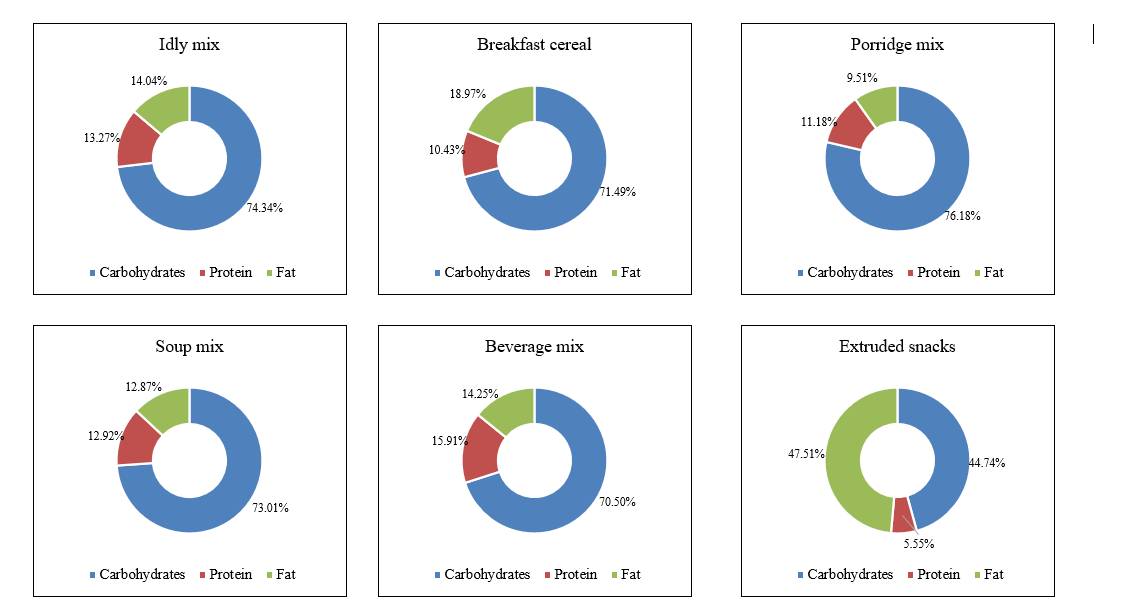

Supplement: S1 Fig — (TIF) [file pone.0314819.s001.tif]

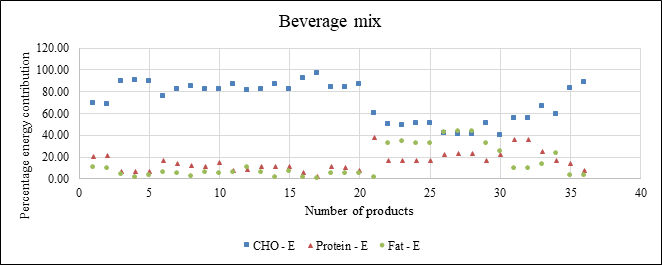

Supplement: S2 Fig — (a). Energy contributed by the macronutrients in commercial products (Idly mix), (b). Energy contributed by the macronutrients in commercial products (Breakfast cereal), (c). Energy contributed by the macronutrients in commercial products (Porridge mix), (d). Energy contributed by the macronutrients in commercial products (Soup mix), (e). Energy contributed by the macronutrients in commercial products (Beverage mix), (f). Energy contributed by the macronutrients in commercial products (Extruded snacks). (ZIP) [file pone.0314819.s002.zip › Fig S2 (e).tif]

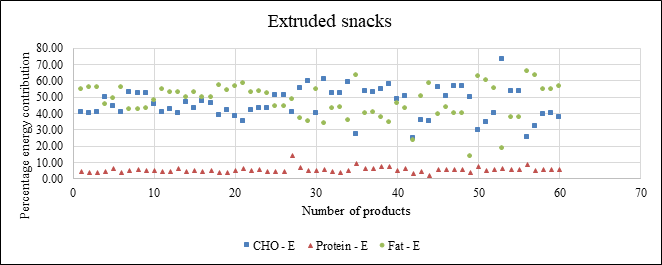

Supplement: S2 Fig — (a). Energy contributed by the macronutrients in commercial products (Idly mix), (b). Energy contributed by the macronutrients in commercial products (Breakfast cereal), (c). Energy contributed by the macronutrients in commercial products (Porridge mix), (d). Energy contributed by the macronutrients in commercial products (Soup mix), (e). Energy contributed by the macronutrients in commercial products (Beverage mix), (f). Energy contributed by the macronutrients in commercial products (Extruded snacks). (ZIP) [file pone.0314819.s002.zip › Fig S2 (f).tif]

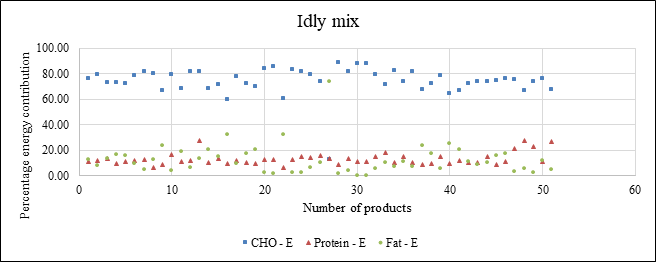

Supplement: S2 Fig — (a). Energy contributed by the macronutrients in commercial products (Idly mix), (b). Energy contributed by the macronutrients in commercial products (Breakfast cereal), (c). Energy contributed by the macronutrients in commercial products (Porridge mix), (d). Energy contributed by the macronutrients in commercial products (Soup mix), (e). Energy contributed by the macronutrients in commercial products (Beverage mix), (f). Energy contributed by the macronutrients in commercial products (Extruded snacks). (ZIP) [file pone.0314819.s002.zip › Fig S2 (a).tif]

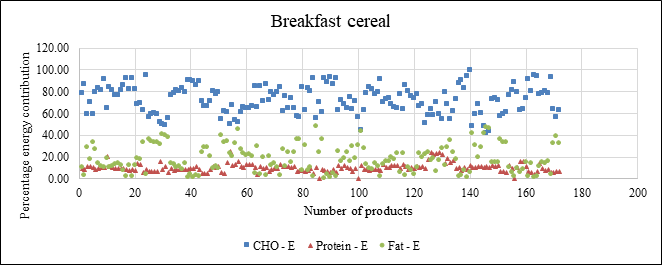

Supplement: S2 Fig — (a). Energy contributed by the macronutrients in commercial products (Idly mix), (b). Energy contributed by the macronutrients in commercial products (Breakfast cereal), (c). Energy contributed by the macronutrients in commercial products (Porridge mix), (d). Energy contributed by the macronutrients in commercial products (Soup mix), (e). Energy contributed by the macronutrients in commercial products (Beverage mix), (f). Energy contributed by the macronutrients in commercial products (Extruded snacks). (ZIP) [file pone.0314819.s002.zip › Fig S2 (b).tif]

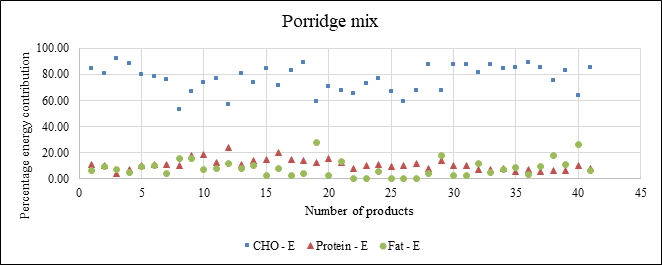

Supplement: S2 Fig — (a). Energy contributed by the macronutrients in commercial products (Idly mix), (b). Energy contributed by the macronutrients in commercial products (Breakfast cereal), (c). Energy contributed by the macronutrients in commercial products (Porridge mix), (d). Energy contributed by the macronutrients in commercial products (Soup mix), (e). Energy contributed by the macronutrients in commercial products (Beverage mix), (f). Energy contributed by the macronutrients in commercial products (Extruded snacks). (ZIP) [file pone.0314819.s002.zip › Fig S2 (c).tif]

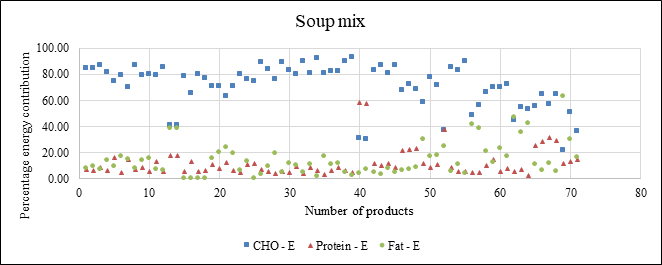

Supplement: S2 Fig — (a). Energy contributed by the macronutrients in commercial products (Idly mix), (b). Energy contributed by the macronutrients in commercial products (Breakfast cereal), (c). Energy contributed by the macronutrients in commercial products (Porridge mix), (d). Energy contributed by the macronutrients in commercial products (Soup mix), (e). Energy contributed by the macronutrients in commercial products (Beverage mix), (f). Energy contributed by the macronutrients in commercial products (Extruded snacks). (ZIP) [file pone.0314819.s002.zip › Fig S2 (d).tif]

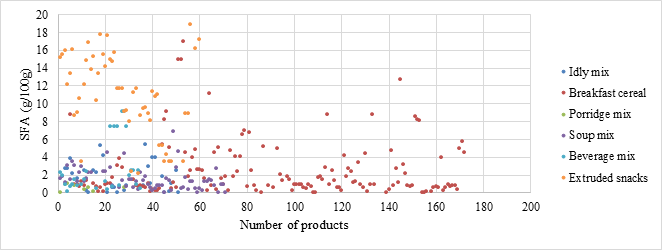

Supplement: S3 Fig — (a). Carbohydrate content of the convenience food products and snacks, (b). Protein content of the convenience food products and snacks, (c). Total fat content of the convenience food products and snacks, (d). Saturated fat content of the convenience food products and snacks, (e). Trans fat content of the convenience food products and snacks, (f). Cholesterol content of the convenience food products and snacks, (g). Dietary fibre content of the convenience food products and snacks, (h). Sugar content of the convenience food products and snacks, (i). Sodium content of the convenience food products and snacks, (j). Energy contributed by the convenience food products and snacks. (ZIP) [file pone.0314819.s003.zip › Fig S3 (d).tif]

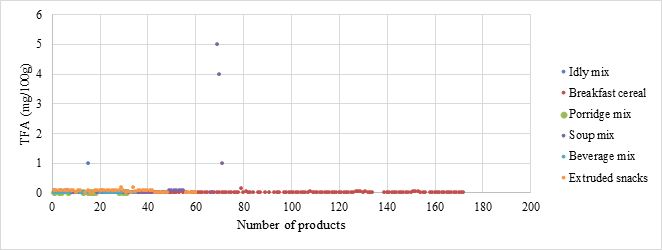

Supplement: S3 Fig — (a). Carbohydrate content of the convenience food products and snacks, (b). Protein content of the convenience food products and snacks, (c). Total fat content of the convenience food products and snacks, (d). Saturated fat content of the convenience food products and snacks, (e). Trans fat content of the convenience food products and snacks, (f). Cholesterol content of the convenience food products and snacks, (g). Dietary fibre content of the convenience food products and snacks, (h). Sugar content of the convenience food products and snacks, (i). Sodium content of the convenience food products and snacks, (j). Energy contributed by the convenience food products and snacks. (ZIP) [file pone.0314819.s003.zip › Fig S3 (e).tif]

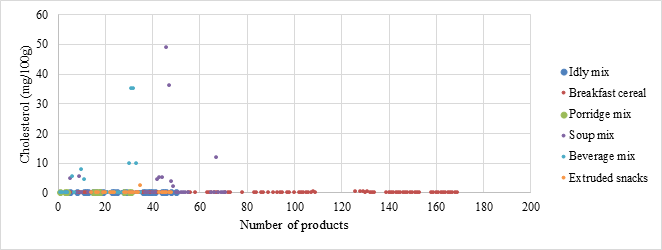

Supplement: S3 Fig — (a). Carbohydrate content of the convenience food products and snacks, (b). Protein content of the convenience food products and snacks, (c). Total fat content of the convenience food products and snacks, (d). Saturated fat content of the convenience food products and snacks, (e). Trans fat content of the convenience food products and snacks, (f). Cholesterol content of the convenience food products and snacks, (g). Dietary fibre content of the convenience food products and snacks, (h). Sugar content of the convenience food products and snacks, (i). Sodium content of the convenience food products and snacks, (j). Energy contributed by the convenience food products and snacks. (ZIP) [file pone.0314819.s003.zip › Fig S3 (f).tif]

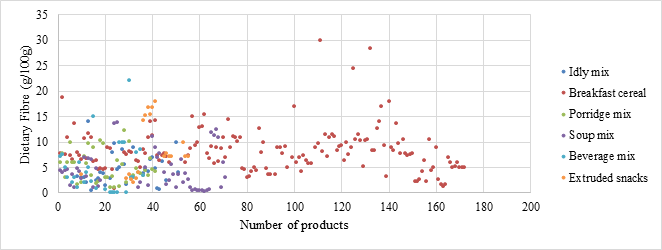

Supplement: S3 Fig — (a). Carbohydrate content of the convenience food products and snacks, (b). Protein content of the convenience food products and snacks, (c). Total fat content of the convenience food products and snacks, (d). Saturated fat content of the convenience food products and snacks, (e). Trans fat content of the convenience food products and snacks, (f). Cholesterol content of the convenience food products and snacks, (g). Dietary fibre content of the convenience food products and snacks, (h). Sugar content of the convenience food products and snacks, (i). Sodium content of the convenience food products and snacks, (j). Energy contributed by the convenience food products and snacks. (ZIP) [file pone.0314819.s003.zip › Fig S3 (g).tif]

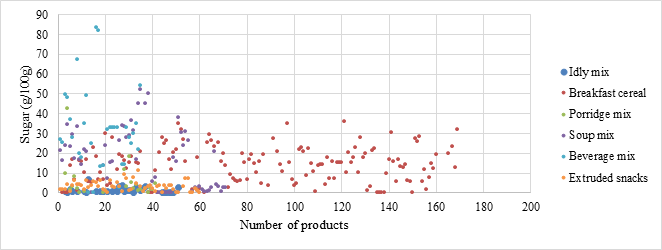

Supplement: S3 Fig — (a). Carbohydrate content of the convenience food products and snacks, (b). Protein content of the convenience food products and snacks, (c). Total fat content of the convenience food products and snacks, (d). Saturated fat content of the convenience food products and snacks, (e). Trans fat content of the convenience food products and snacks, (f). Cholesterol content of the convenience food products and snacks, (g). Dietary fibre content of the convenience food products and snacks, (h). Sugar content of the convenience food products and snacks, (i). Sodium content of the convenience food products and snacks, (j). Energy contributed by the convenience food products and snacks. (ZIP) [file pone.0314819.s003.zip › Fig S3 (h).tif]

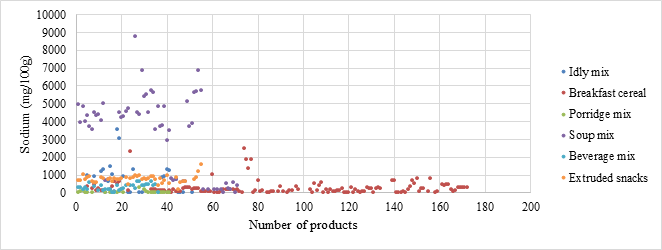

Supplement: S3 Fig — (a). Carbohydrate content of the convenience food products and snacks, (b). Protein content of the convenience food products and snacks, (c). Total fat content of the convenience food products and snacks, (d). Saturated fat content of the convenience food products and snacks, (e). Trans fat content of the convenience food products and snacks, (f). Cholesterol content of the convenience food products and snacks, (g). Dietary fibre content of the convenience food products and snacks, (h). Sugar content of the convenience food products and snacks, (i). Sodium content of the convenience food products and snacks, (j). Energy contributed by the convenience food products and snacks. (ZIP) [file pone.0314819.s003.zip › Fig S3 (i).tif]

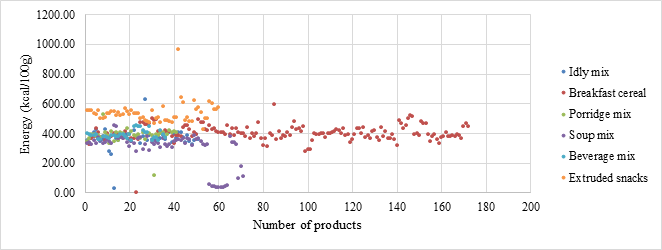

Supplement: S3 Fig — (a). Carbohydrate content of the convenience food products and snacks, (b). Protein content of the convenience food products and snacks, (c). Total fat content of the convenience food products and snacks, (d). Saturated fat content of the convenience food products and snacks, (e). Trans fat content of the convenience food products and snacks, (f). Cholesterol content of the convenience food products and snacks, (g). Dietary fibre content of the convenience food products and snacks, (h). Sugar content of the convenience food products and snacks, (i). Sodium content of the convenience food products and snacks, (j). Energy contributed by the convenience food products and snacks. (ZIP) [file pone.0314819.s003.zip › Fig S3 (j).tif]

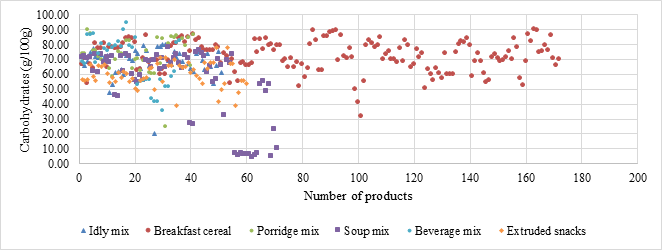

Supplement: S3 Fig — (a). Carbohydrate content of the convenience food products and snacks, (b). Protein content of the convenience food products and snacks, (c). Total fat content of the convenience food products and snacks, (d). Saturated fat content of the convenience food products and snacks, (e). Trans fat content of the convenience food products and snacks, (f). Cholesterol content of the convenience food products and snacks, (g). Dietary fibre content of the convenience food products and snacks, (h). Sugar content of the convenience food products and snacks, (i). Sodium content of the convenience food products and snacks, (j). Energy contributed by the convenience food products and snacks. (ZIP) [file pone.0314819.s003.zip › Fig S3 (a).tif]

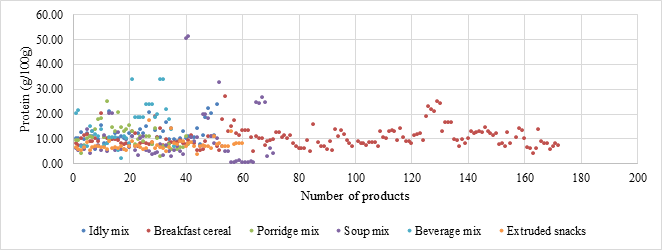

Supplement: S3 Fig — (a). Carbohydrate content of the convenience food products and snacks, (b). Protein content of the convenience food products and snacks, (c). Total fat content of the convenience food products and snacks, (d). Saturated fat content of the convenience food products and snacks, (e). Trans fat content of the convenience food products and snacks, (f). Cholesterol content of the convenience food products and snacks, (g). Dietary fibre content of the convenience food products and snacks, (h). Sugar content of the convenience food products and snacks, (i). Sodium content of the convenience food products and snacks, (j). Energy contributed by the convenience food products and snacks. (ZIP) [file pone.0314819.s003.zip › Fig S3 (b).tif]

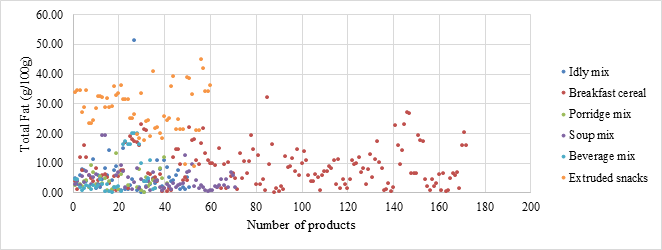

Supplement: S3 Fig — (a). Carbohydrate content of the convenience food products and snacks, (b). Protein content of the convenience food products and snacks, (c). Total fat content of the convenience food products and snacks, (d). Saturated fat content of the convenience food products and snacks, (e). Trans fat content of the convenience food products and snacks, (f). Cholesterol content of the convenience food products and snacks, (g). Dietary fibre content of the convenience food products and snacks, (h). Sugar content of the convenience food products and snacks, (i). Sodium content of the convenience food products and snacks, (j). Energy contributed by the convenience food products and snacks. (ZIP) [file pone.0314819.s003.zip › Fig S3 (c).tif]
