# Supplementary material for: Assessment of front and back of pack nutrition labels of selected convenience food products and snacks available in the Indian market
Source: PLoS One. 2024 Dec 6;19(12):e0314819. doi: 10.1371/journal.pone.0314819 (PMC11623555; doi:10.1371/journal.pone.0314819)
Supplement: Attachment — Submitted filename: Response to Reviewers.docx [file pone.0314819.s005.docx]

13.11.2024

From

Dr. Shobana Shanmugam

Senior Scientist and Head

Department of Diabetes Food Technology

Madras Diabetes Research Foundation, Chennai

To

The Editor in Chief

PLOS One

Subject: Revision of manuscript (PONE-D-24-46848) – reg.,

Dear Editor in Chief,

We are submitting the revised manuscript **PONE-D-24-46848** entitled **‘Assessment of front and back of pack nutrition labels of selected convenience food products and snacks available in the Indian market’**. The authors are grateful to the reviewers for their insightful comments and critical assessment on the paper. The manuscript has been revised according to the suggestions of the reviewers. The queries of the reviewers have been addressed and the detailed response has been enclosed with the letter. As recommended, we have submitted the marked-up copy and unmarked version of the revised manuscript. We have revised the manuscript to meet the PLOS ONE journal’s style requirements and submitted the ‘minimal data set’ consisting of the data analysed in the study.

Thank you for your time and support.

Thanking you and with regards,

Shobana Shanmugam

**Response to Reviewers**

**Reviewer #1: Comments**

1. **Abstract: - The abbreviations FSSAI and INFORMAS should be defined upon their first mention.**

*Response:* Thank you for the suggestion. The abbreviations for FSSAI and INFORMAS has been included in the abstract (Page no: 2, Line 8 and Lines 14 – 15).

2. **Introduction: - I suggest standardizing the _indentation_ of the paragraphs.**

*Response:* Thank you for pointing this out. The indentations have been standardized throughout the manuscript. The manuscript has been revised to meet the PLOS ONE's style requirements.

3. **Methods in Data Analysis: - I recommend removing the quotation marks around the terms, as it is clear that these were the terms used, making the quotation marks unnecessary.**

*Response:* The authors and have removed the quotation marks around the claims throughout the manuscript (Page no: 5, Lines 83 – 84).

4. **Results: Table 1a:**
   - **Please include a legend below the table with definitions for SFA, RDA, and GI.**
   - **In the legend, only the abbreviations can be used within the table.**
   - **Include the information: Source: FSSAI, 2018, in the legend.**

*Response:* Thank you for the suggestion. The abbreviations and definitions for SFA, RDA and GI has been included in the legend of Table 1a. In addition, the information about the source has also been included in the legend of Table 1a (Page no: 7, Lines 95 – 100).

5. **Table 1b: - Include the information: Source: FSSAI, 2018, in the legend.**

*Response:* Thank you for the suggestion. The information about the source has been included in the legend of Table 1b (Page no: 2, Lines 103 – 104).

6. **Line 111 (referring to Table 3): - I suggest removing the quotation marks around the claims, as it is clear that these were the terms used, making the quotation marks unnecessary.**

*Response:* The authors agree with the suggestion and have removed the quotation marks around the claims throughout the manuscript (Page no: 11, Lines 122 – 137).

7. **Table 3: - Please include a legend below the table with a definition for GI. Include the information: Source: FSSAI, 2018, in the legend.**

*Response:* Thank you for pointing it out. The definition of Glycemic Index (GI) has been included in the legend of Table 1a. Hence, only the details regarding the source have been included in the legend of Table 3 (Page no: 13, Lines 141 – 142).

8. **Line 132 (referring to Fig. 2): - I suggest removing the quotation marks around the claims, as it is clear that these were the terms used, making the quotation marks unnecessary.**

*Response:* We agree and have removed the quotation marks around the claims throughout the manuscript (Page no: 14, Lines 145 – 149).

9. **Line 158 (referring to Fig. 3): - I suggest removing the quotation marks around the claims, as it is clear that these were the terms used, making the quotation marks unnecessary.**

*Response:* The authors agree with the suggestion and have removed the quotation marks around the claims throughout the manuscript (Page no: 18, Lines 170 – 172 and Lines 179).

10. **Discussion: - In the first paragraph, I suggest reformulating it to include only data from this research as a brief summary, before contextualizing in the following paragraphs.**

*Response:* Thank you. The first paragraph of the discussion section has been rephrased accordingly to emphasize your suggestion (Page no: 19).

11. **Conclusion: - I recommend including the percentage in the section between lines 274 and 280. I suggest removing the quotation marks around the claims, as it is clear that these were the terms used, making the quotation marks unnecessary.**

*Response:* The authors agree with the suggestion and have removed the quotation marks around the claims throughout the manuscript. The percentage of products was included in the conclusion (Page no: 23, Lines 306 – 311).

12. **Fig. 1: - I suggest improving the image quality. If abbreviations are used, I recommend providing them in the form of a legend, rather than combining definitions and abbreviations in the image.**

*Response:* Thank you. The image quality of Fig. 1 has been enhanced to meet the PLOS ONE's style requirements.

13. **Fig. 2: - I suggest improving the image quality. If abbreviations are used, I recommend providing them in the form of a legend, rather than combining definitions and abbreviations in the image.**

*Response:* The image quality of Fig. 2 has been enhanced to meet the PLOS ONE's style requirements.

14. **Fig. 3: - I suggest improving the image quality.**

*Response:* Thank you for pointing this out. The image quality of Fig. 3 has been enhanced to meet the PLOS ONE's style requirements.

**Reviewer #2: Comments**

1. **The manuscript examines only six categories of packaged foods, which may not be representative of the broader range of convenience foods available in the Indian market. This restricts the generalizability of findings to all packaged foods and limits insight into other widely consumed food categories, such as snacks and sweets specific to regional diets.**

*Response:* The present study was part of a bigger study which aimed to develop convenience foods (the products evaluated in the market survey) with lower fat, higher protein and fibre content and in few products with lower glycemic index. The information generated in the market survey was used for product development. Hence, the market survey included selected products.

2. **Although the traffic light system is a well-recognized method, it has limitations, particularly in the Indian context where consumer understanding of this labelling system might be low. The study does not mention whether the system was validated for Indian consumers or if alternative methods that might be more culturally or regionally relevant were considered. Additionally, the traffic light system only focuses on fat, saturated fat, and sugar content, omitting other potentially relevant nutrients like sodium, which are significant in packaged foods and may affect overall health.**

*Response:* Thank you for pointing this out and this a very valuable comment. A cross-sectional study with a quasi-experimental design was conducted by National Institute of Nutrition, Hyderabad to examine the food label reading habits of the participants along with their views on the acceptability of various formats of front of pack nutritional label in India^1^. The results of the study concluded that the number of participants agreed the traffic light labelling helped them to identify healthy and unhealthy food was 85% and 83.4% respectively. Around 89.9% of participants declared the traffic light labelling is easier to understand. Hence, we have used traffic light system to categorize the food products. However, we couldn’t include salt content which is a part of traffic light system categorization as in Indian products, sodium levels are declared rather than sodium chloride content. Sodium is contributed by not only sodium chloride but also food ingredients, several other food additives, preservatives making it difficult to compute the sodium chloride values. The explanation has been included in the revised manuscript (Page no: 22 & 23, Lines 286 – 299).

3. **The paper does not clarify if nutritional content was analyzed per 100 grams or based on serving sizes. This can affect how “healthy” or “less healthy” a product is perceived, as nutrient density can vary widely depending on portion size. A more accurate assessment would consider both serving size and per 100-gram analysis to better reflect actual consumption patterns.**

*Response:* Thank you for the valuable comment and suggestion. However, as explained in the manuscript all the descriptive statistics of the products were provided for 100g of the product. The FSSAI nutritional claim and traffic light system has provided nutrient thresholds for 100g of the product. Hence the nutritional content of the products was analyzed only for 100g of the product. The serving size information hasn’t been declared in many products and hence the same was not included in the analysis. We have mentioned the same in the data collection methodology (Page no: 4, Lines 64 – 72, Page no: 5, Lines 86 – 87), table 1a (Page no:6, Line 94), table 1b (Page no:7, Line 101).

4. **While the study suggests reformulation for extruded snacks to reduce fat content, it does not address the broader challenges of reformulation in the Indian food industry, such as taste preferences, preservation requirements, and cost implications. The recommendation could be further enhanced by discussing the feasibility of reformulation in alignment with market demands and cost-effectiveness for manufacturers, which is crucial for practical implementation.**

*Response:* Thank you for the valuable inputs. Yes, we agree, more studies on the utilization of innovative processing technologies, product reformulation and alternative approaches for incorporating seasoning in extruded snacks with lower fat content will be helpful. However, more trials and sensory acceptability studies, process, product economics needs to be evaluated and matched with the market demand. The aforementioned aspect has been included in the discussion section of the revised manuscript (Page no: 21,22; Lines 265 – 268).

5. **The manuscript assesses product labels without considering consumer comprehension or response to nutritional information, which is essential to understand how effective labelling is in guiding healthier choices. Including consumer surveys or focus groups would add valuable insights into whether nutritional labels influence purchasing behaviour or if additional educational interventions are necessary.**

*Response:* Thank you for the valuable suggestion. However, the objective of the current survey was to understand the information on the nutritional, ingredient composition, nutritional content claims (declared on the packs) and their compliance according Food Safety Standards Authority of India (FSSAI), categorization of the foods as per Food Standards Agency (FSA), UK for convenience foods. In future, we would like to take up such studies to understand the influence of food labelling on consumers in guiding healthier choices.

6. **While the study references FSSAI (Food Safety and Standards Authority of India) regulations, it does not provide a comparison with** **international standards or guidelines, such as those from the World Health Organization (WHO) or other national regulatory bodies. This could provide perspective on how Indian regulations align with or diverge from global best practices and help in identifying potential areas for regulatory improvement.**

*Response:* Thank you for pointing it out. The WHO guidelines have provided the threshold information for different categories of products. However, the guidelines didn’t include standards for *idly mix* and porridge mix. Many other international standards didn’t include the different categories of foods available in the Indian market. Hence for the current study, we have considered only the national guidelines (FSSAI). The limitation has been included in the revised manuscript (Page no: 22, Lines 282 – 285).

7. **Finally, the paper finds an 80.5% compliance rate for nutrient content claims but does not explore why the remaining 19.5% did not comply. It would be helpful to understand whether non-compliance was due to inaccurate claims, misleading language, or other regulatory issues, as this could highlight areas for improved regulatory enforcement and consumer protection.**

*Response:* Thank you for the valuable comment. The non-compliance of the nutritional content claims was due to inaccurate claims and error/missing nutritional information which is required to verify the claims. We have mentioned the reasons for non-compliance of the claims in the revised manuscript (Page no: 18; Lines 185 – 187).
